# Developing a Thai national critical care allocation guideline during the COVID-19 pandemic: a rapid review and stakeholder consultation

**DOI:** 10.1186/s12961-021-00696-z

**Published:** 2021-03-31

**Authors:** Aniqa Islam Marshall, Rachel Archer, Woranan Witthayapipopsakul, Kanchanok Sirison, Somtanuek Chotchoungchatchai, Pisit Sriakkpokin, Orapan Srisookwatana, Yot Teerawattananon, Viroj Tangcharoensathien

**Affiliations:** 1 International Health Policy Program, Ministry of Public Health, Nonthaburi, Thailand; 2 Health Intervention and Technology Assessment Program, Ministry of Public Health, Nonthaburi, Thailand; 3 The National Health Commission Office, Ministry of Public Health, Nonthaburi, Thailand; 4 Saw Swee Hock School of Public Health (SSHSPH), National University of Singapore, Singapore, Singapore

**Keywords:** COVID-19, Pandemic, Prioritization, Rationing, Critical care resource, Resource allocation, Stakeholder consultation, Guideline development, Rapid guidelines, Thailand

## Abstract

**Background:**

At the height of the COVID-19 pandemic, Thailand had almost depleted its critical care resources, particularly intensive care unit (ICU) beds and ventilators. This prompted the necessity to develop a national guideline for resource allocation. This paper describes the development process of a national guideline for critical resource allocation in Thailand during the COVID-19 pandemic.

**Methods:**

The guideline development process consisted of three steps: (1) rapid review of existing rationing guidelines and literature; (2) interviews of Thai clinicians experienced in caring for COVID-19 cases; and (3) multi-stakeholder consultations. At steps 1 and 2, data was synthesized and categorized using a thematic and content analysis approach, and this guided the formulation of the draft guideline. Within step 3, the draft Thai critical care allocation guideline was debated and finalized before entering the policy-decision stage.

**Results:**

Three-order prioritization criteria consisting of (1) clinical prognosis using four tools (Charlson Comorbidity Index, Sequential Organ Failure Assessment, frailty assessment and cognitive impairment assessment), (2) number of life-years saved and (3) social usefulness were proposed by the research team based on literature reviews and interviews. At consultations, stakeholders rejected using life-years as a criterion due to potential age and gender discrimination, as well as social utility due to a concern it would foster public distrust, as this judgement can be arbitrary. It was agreed that the attending physician is required to be the decision-maker in the Thai medico-legal context, while a patient review committee would play an advisory role. Allocation decisions are to be documented for transparency, and no appealing mechanism is to be applied. This guideline will be triggered only when demand exceeds supply after the utmost efforts to mobilize surge capacity. Once implemented, it is applicable to all patients, COVID-19 and non-COVID-19, requiring critical care resources prior to ICU admission and during ICU stay.

**Conclusions:**

The guideline development process for the allocation of critical care resources in the context of the COVID-19 outbreak in Thailand was informed by scientific evidence, medico-legal context, existing norms and societal values to reduce risk of public distrust given the sensitive nature of the issue and ethical dilemmas of the guiding principle, though it was conducted at record speed. Our lessons can provide an insight for the development of similar prioritization guidelines, especially in other low- and middle-income countries.

## Background

The coronavirus disease 2019 (COVID-19) outbreak was declared a global pandemic by WHO on 11 March 2020 [1]. To date, there have been almost 15 million confirmed cases and over 600 000 deaths [2]. Across the world, COVID-19 has overwhelmed healthcare systems and their capacity to respond. There have been extensive reports of inadequate supplies of personal protective equipment for healthcare workers and shortages of intensive care beds and ventilators in many countries [3]. The surplus in demand exceeding the availability of healthcare resources has led to the unavoidable rationing of medical equipment and interventions, notably critical care resources which are challenging to expand in a short time [3–7]. As a result, several countries have been compelled to develop national resource allocation guides, specific to COVID-19 and country context [8–12].

Thailand reported the first case of the virus outside of China in early January 2020 [13, 14]. By the end of January 2020, the first known person-to-person transmission in the country was documented, and a total of 3255 cases and 58 deaths were confirmed as of 21 July 2020 [15].

Critical resources matched with the number of daily reported cases inform policy-makers on potential resource gaps. As of April 2020, in Bangkok, there were 105 public and private hospitals for COVID-19 patients requiring critical care with a total of 1978 specialized beds; including 120 beds in airborne infection isolation rooms (AIIR) in intensive care units (ICUs), 108 in modified AIIRs, 1056 in isolation rooms and 694 in cohort wards specially designed to accommodate large numbers of less severe cases. Additionally, 603 hospital rooms were allocated to provide step-down care. Outside of Bangkok, there were 4955 ICU beds, 319 AIIR ICUs, 742 modified AIIRs and 2497 isolation rooms and 3031 cohort wards. Nation-wide, there are 3000 ventilators in Bangkok and 10 184 in the remaining provinces. Table 1 presents critical care resources for treatment of severe COVID-19 patients. As a response to the resource need for COVID-19 at the epicentre, in Bangkok, critical resources were updated on a dashboard and reported daily to the emergency operations centre.

**Table 1:** Critical care resources for treatment of severe COVID-19 patients

	ICU beds		Ventilators	
	Number	Per million population	Number	Per million population
Bangkok	1 978	198	3 000	300
All other 76 provinces	4 955	88	10 184	182
	6 933	105	13 184	200

The Ministry of Public Health (MOPH) maximized, expanded and mobilized critical care resources from all public and private health facilities throughout the country, and earmarked 10% of all ICU beds for COVID-19 patients, while keeping the remaining for non-COVID-19 critical patients [16]. However, at the peak of the epidemic (22 March–3 April), there were between 91 to 188 new cases per day, causing the number of available ICU beds and AIIRs in the country to almost reach its maximum capacity, placing significant strain on health facilities [15, 16]. Recognizing that the current critical care resource capacity may reach its threshold, the development of a national guideline for resource allocation was deemed necessary.

In the context of developing a guideline during a public health emergency, such as the COVID-19 pandemic, the standard time frame of 6 months to 3 years for guideline development is not appropriate; therefore, a rapid guideline development approach is needed [17, 18]. This paper aims to describe the rapid development of a critical care resource allocation guideline in Thailand during a public health emergency and share lessons learnt that may serve as a useful example for other countries.

## Methods

In response to the COVID-19 pandemic, researchers from the Health Intervention and Technology Assessment Program (HITAP) and International Health Policy Program (IHPP), the two health systems and policy research institutes of the MOPH serving as the technical secretariat of the MOPH Intelligence Unit (MIU), were tasked by the MIU with developing a guideline for prioritizing critical care resources. The two units partnered with the Thai National Health Commission Office (NHCO) to capitalize on the NHCO’s capacity in convening the annual National Health Assembly, and its network with different stakeholders and civil society organizations (CSOs) [19]. Together, the three bodies formed a technical team to lead the guideline development. To develop an evidence-informed guide within a short time frame, the technical team designed three steps for guideline development: (1) rapid review of existing rationing guidelines; (2) interviews of clinicians experienced in caring for COVID-19 cases; and (3) multi-stakeholder consultations.

### Rapid literature review

The main objective of the literature review was to identify existing national and international guidelines, recommendations and frameworks on critical care resource allocation, patient triage policies and resource management during pandemic situations. A preliminary search on PubMed database using the keywords “rationing” OR “allocation” OR “COVID” yielded few academic papers at the time of search, with mostly commentaries on ethical principles, challenges and recommendations rather than allocation guides as intended, due to most usually being published as grey literature [20, 21]. Therefore, the technical team hand-searched from the International Society for Priorities in Health website, which established a COVID-19 platform comprising academic literature on priority setting and guidelines specific or applicable to COVID-19 from various sources [17, 18, 22]. Guidelines were screened for relevance, and 11 guides from seven countries and one international agency were selected, based on an inclusion criterion of being published between 2010 and 2020 and exclusion criteria of not being available in a language that could be readily translated by the technical team.

### Key informant interview

This process aimed to help the technical team understand the current practices on ICU admissions, patient triage methods and critical care resource allocation in Thailand as well as the need for and feasibility of implementing a new guideline. We invited four intensive care experts working in public and private hospital ICUs. As COVID-19 peaked in March and April, which demanded their clinical services, only two ICU physicians working in public hospitals were able to participate in the interviews through telephone calls. Note that physicians are the prime decision-makers on the use of ICU beds and other critical care resources.

### Stakeholder consultation

As public health emergencies pose several ethical, social and legal dilemmas, the stakeholder consultation aimed to incorporate the views and expertise of relevant groups to ensure the guideline’s feasibility and acceptability to Thai society [17]. Broad-base stakeholder groups were identified by the technical team, aiming for those with a technical expertise in a relevant field and also a sound understanding of health policy processes. To ensure full deliberation by stakeholders, two rounds of half-day consultations were convened, the first for medicine and medical law stakeholders and the second policy-makers and social science stakeholders (Table 2). Documents pertaining to the details of the proposed guideline developed by the technical team were shared with all stakeholders prior to the consultation. Participants attended either in person or via teleconference call.

**Table 2:** Stakeholders involved in multi-stakeholder consultation

Stakeholder group	Gender		Attendance		Total
	Male	Female	Teleconference	In person	
Round 1: medicine and medical law stakeholders					
Palliative care specialist	1	1	2	0	2
Respiratory care specialist	1	0	1	0	1
Epidemiologist	0	1	1	0	1
Emergency medicine specialist	1	0	1	0	1
Obstetrician gynaecologist	1	0	1	0	1
Paediatrics/family medicine	0	1	1	0	1
Medical lawyer	2	1	1	2	3
Total	6	4	8	2	10
Round 2: policy-makers and social science stakeholders					
Policy-maker	2	0	0	2	2
Medical anthropologist	1	0	1	0	1
Civil society organization representative	0	3	3	0	3
Religious leader/scholar	4	0	4	0	4
Public communications specialist	1	0	1	0	1
Total	8	3	9	2	11

The consultation consisted of a short presentation of the proposed guideline and courses of action to consider, followed by an open discussion. At each round of consultation, an open forum approach was utilized and moderated by a senior member of the technical team to allow for the incorporation of all views and to reach consensus on key issue areas. The first round of consultation informed a revised guideline presented during the second round. The final text, after consultation, was circulated to all stakeholders for their further written comment or endorsement.

### Data analysis and synthesis

All included documents from the rapid literature review were reviewed and key data extracted using a data extraction form. Information from each interview was summarized by the interviewee, and key issue areas were identified through discussion among the technical team. Immediately following each consultation, the technical team summarized and categorized all issues into similar thematic areas using a thematic and content analysis approach.

## Results

### Existing guidelines

Data extracted from the rapid review of 11 included guides are summarized in Table 3. Content was extracted and grouped into the following key themes: guiding principles, target patients, prioritization criteria, decision-maker and process, and implementation.

**Table 3:** Data extraction of existing guidelines on critical care resource allocation

Country/setting	Guiding principles	Target patients	Prioritization criteria	Decision-making process	Implementation conditions
1. Austria [40]	Ethical principles of justice, beneficence, well-being, autonomy	All patients needing critical care	Comorbidities	Decision-maker: intensive care specialist Process: consultation with designated experts and patients and relatives Time of decision-making: –	Health resource demand exceeds supply
2. Belgium [11]	First come, first served Randomization	All patients needing critical care	First-come, first served; medical urgency; cognitive impairment; patient age; comorbidities	Decision-maker: Team of healthcare professionals Process: consultation with experts (technical, nursing, etc.)/patient’s general practitioner Time of decision-making: upon admission with daily reassessment	Health resource demand exceeds supply
3. Germany [12]	Clinical success	All patients needing critical care	Comorbidities	Decision-maker: team of healthcare professionals Process: consultation with experts (technical, nursing, etc.)/patient’s general practitioner Time of decision-making: upon admission	Health resource demand exceeds supply
4. Italy [23]	Greatest life expectancy	All patients needing critical care	Patient age; Comorbidities	Decision-maker: healthcare staff with patients, proxies + others (ethics committees) Process: consultation with designated experts and patients/relatives Time of decision-making: upon admission with daily reassessment	Health resource demand exceeds supply
5. Switzerland [9]	Beneficence Non-maleficence Respect for autonomy Equity	All patients needing critical care	Patient age; comorbidities	Decision-maker: team of healthcare professionals Process: consultation with ethics committee/team Time of decision-making: upon admission with reassessment every 2–3 days	
6. United Kingdom (NHS) [10]	Clinical success	All patients needing critical care	Clinical frailty; comorbidities	Decision-maker: team of healthcare professionals Process: consultation with experts (technical, nursing, etc.)/patient’s general practitioner Time of decision-making: upon admission	
7. United Kingdom (BMA) [41]	Promote safe and effective patient care as far as possible in the circumstances	All patients needing critical care		Decision-maker: team of healthcare professionals Time of decision-making: upon admission	
8. United States (Hastings Center) [42]	Promote equality and equity in distribution of the risks and benefits in society	All patients needing critical care		Decision-maker: healthcare staff with patients, proxies and others (ethics committees) Process: consultation with designated experts and patients/relatives Time of decision-making: upon admission	
9. United States (New York) [24]	Save the most lives	All patients needing critical care	First come, first served; randomization; social usefulness; patient age; comorbidities; Sequential Organ Failure Assessment	Decision-maker: nominated triage officer or triage committees Process: consultation with experts (technical, nursing, etc.)/patient’s general practitioner Time of decision-making: upon admission with reassessment after 48 and 120 h	Health resource demand exceeds supply
10. United States (Pittsburgh) [25]	Duty to care Duty to steward resources to optimize population health Distributive and procedural justice Transparency	All patients needing critical care	Patient age; comorbidities	Decision-maker: nominated triage officer or triage committees Process: consultation with designated experts Time of decision-making: upon admission	Health resource demand exceeds supply
11. International (WHO) [43]	Utility and equity, on the basis of health-related considerations	All patients needing critical care		Decision-maker: intensive care specialist Time of decision-making: upon admission	Health resource demand exceeds supply

*NHS* National Health Service, *BMA* British Medical Association

### Guiding principles and prioritization criteria

**Fig. 1:**
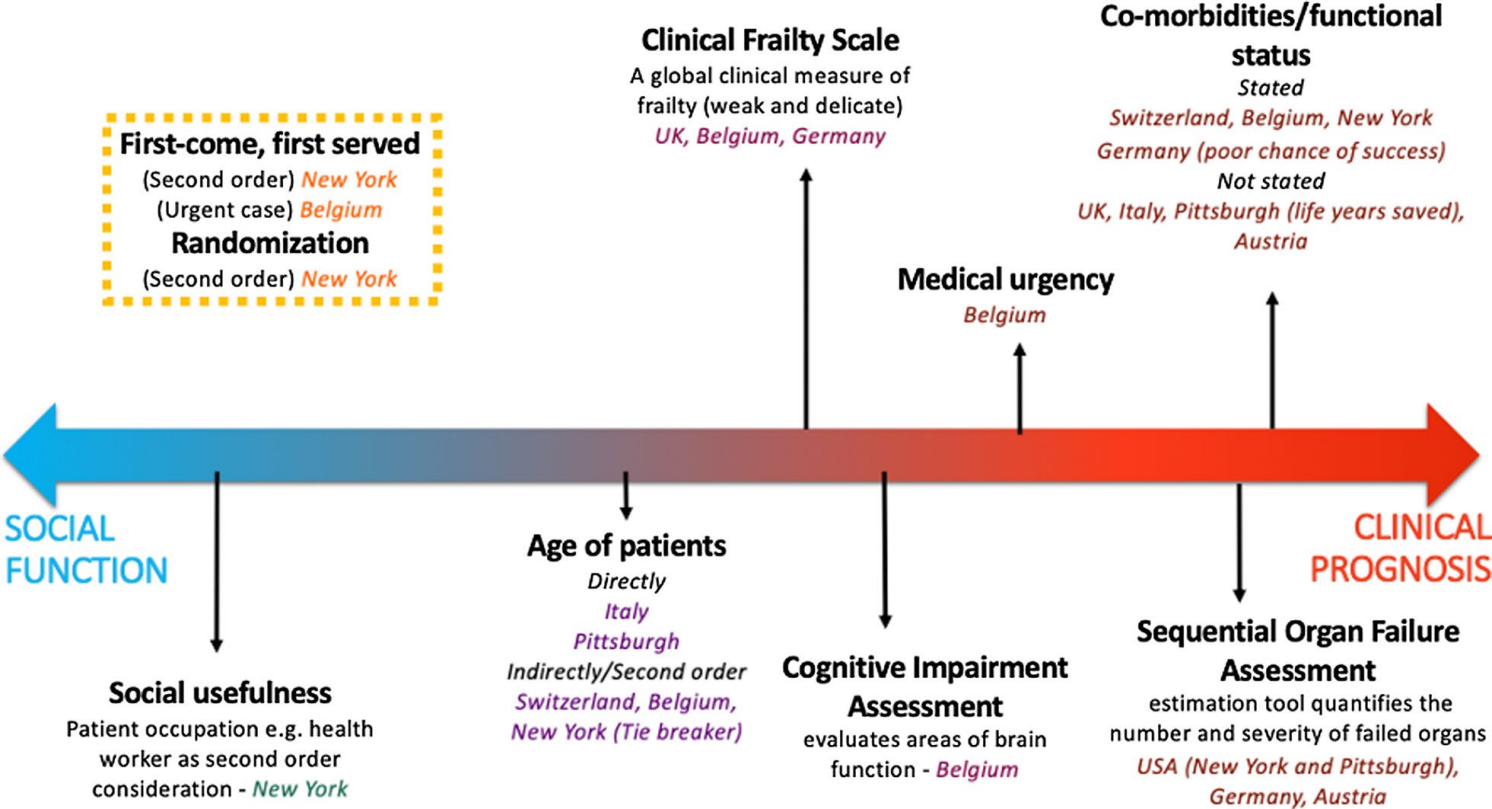
Prioritization criteria scale: from social function to clinical prognosis

The prioritization criteria and tools applied for each guide were positioned by the authors on a scale ranging from social function, which promotes and rewards instrumental value or benefits to others, to clinical prognosis, which gives value to clinical success, the number of lives and life-years that can be saved (see Fig. 1). Social usefulness, such as patient occupation, was applied only for the New York guide. Italy and Pittsburgh both utilized patient age, and it was indirectly assessed in the guides from Switzerland, Belgium and New York. Clinical frailty, which involves assessing the status of cognitive and physical function, is applied in the United Kingdom, Belgium and Germany. Cognitive impairment assessment, measuring brain function, and the patients’ medical urgency were criteria only applied in the Belgium guide. The Sequential Organ Failure Assessment (SOFA), a tool used to estimate and quantify the number and severity of potential organ failure, was used in New York, Pittsburgh, Germany and Austria. Comorbidities, such as prior medical conditions were stated as criteria in the guides from Switzerland, Belgium, New York and Germany, and indirectly considered for the United Kingdom, Italy, Pittsburgh and Austria guides. Additionally, a first-come, first-served method applied in New York and Belgium, as well as randomization, utilized in New York, were excluded from the scale. This wide-ranging scale obtained from the rapid literature review reflects differing societal and cultural values given on allocation criteria.

#### Target patients and guideline application

All guides indicated that the prioritization criteria were to be applied to all patients requiring critical care resources, which includes both COVID-19 and non-COVID-19 patients, to ensure that everyone had the same chance of accessing the scarce resources. The prioritization criteria for most guides was recommended to be applied on admission to ICUs. Additionally, some guides recommended reassessment following ICU admission: daily for the Belgian and Italian guides, every 2–3 days for the Swiss guide and every 48 to 120 h for the New York ventilator allocation guide.

### Decision-making process

The recommendation for the primary decision-maker for the guidelines differed. Decisions to be made by a team of healthcare professionals involved in the patient’s care was suggested in Switzerland, United Kingdom, Belgium and Germany. While United States guides from Pittsburgh and New York recommended the formation of a triage committee or nominating a triage officer to make decision, sparing those involved in direct patient care. Similarly, the Austrian and WHO guides suggested the nomination of an intensive care specialist as the decision-maker. In addition to the primary decision-maker, most guides recommended consultations to be made with an ethics committee (Switzerland, Germany, Austria and United States), technical or designated experts (United Kingdom, Belgium, Germany, New York, Pittsburgh, Italy) or with patients and/or relatives (Italy, Austria, United States). Three out of the 11 guides also provided information regarding the process of appeals against decisions made for the patient. The WHO guidance stated that mechanisms to resolve disputes are necessary. The Pittsburgh guide recommended the formation of a triage review committee to review the appeal using majority vote, while the Italian guide suggested appeals be reviewed by designated experts or regional health centres. Decision-making processes varied between settings, reflecting diverse medico-legal and clinical practices across the world.

### Implementation

Although all guidelines were developed for the pandemics or outbreaks, only the guides from Belgium, Germany, Pittsburgh, New York, Italy, Austria and WHO clearly specified their application only upon demand exceeding supply. No guides stated legal mechanisms for enforcement; instead, all were voluntary and nonbinding recommendations. This ensured the guides could be flexible and adapted to suit the situation and context of each health facility and the changes in clinical data. However, the New York guideline discussed the concern of lack of statutory protection for healthcare workers and institutions.

### Current practices in Thailand

The key informant interview confirmed that no protocols or guidelines on allocating critical care resources currently exist or are being applied in Thailand, though they agreed that such guideline would be useful to ensure a consistent approach across patients and facilitate patient referral across hospitals during public health emergency. The decisions on allocating critical care resources are primarily made by ICU doctors, usually in consensus and based on several qualitative and quantitative factors including medical urgency, SOFA score and comorbidities such as the Charlson Comorbidity Index. In addition to clinical prognosis, patients’ cognitive function may be evaluated through relatives; examples provided included the Modified Informant Questionnaire on Cognitive Decline in the Elderly (modified IQ CODE) and Functional Assessment Staging Test (FAST). It was maintained that cognitive impairment assessments are not usually undertaken by patients themselves given their critical conditions, though discussions with relatives and caregivers may be utilized by physicians to assess the patient’s cognitive function. While reassessment following initial admission may be necessary, many doctors are reluctant to step down treatment or withdraw care except upon the patient’s prior consent not to treat.

In addition to lack of allocation protocols, advanced care plans are not routinely practised, and as attending ICU physicians are on rotation, adequate communication between ICU doctors and patient relatives is lacking. Overall, key informants expressed the need for the development of a national guideline, especially in the public health emergency, and also stressed the importance of the guideline being endorsed by the Thai Medical Council and various Royal Colleges of physicians to ensure successful implementation.

### Proposed draft guideline

Based on the rapid review and key informant interviews, a draft guideline was developed by the technical team to be proposed at the stakeholder consultations. At both rounds of consultations, the technical team proposed four areas for discussion: (1) criteria for patient assessment in allocating resources, (2) decision-maker and decision-making process, (3) appeal and documentation mechanisms and (4) process of implementation and enforcement.

#### Prioritization criteria

The proposed criteria comprised three orders to be applied sequentially to break ties in decisions between patients with the same level of priority. In the context of limited critical care resources, the first-order criteria aimed to assess patients based on short-term clinical prognosis and maximize the health outcomes. Four assessment tools, which would be chosen based on applicability for each health facility setting, were put forward: (1) Charlson Comorbidity Index, (2) SOFA, (3) Clinical Frailty Scale (CFS) and (4) cognitive impairment assessment. The second-order criterion was the number of potential life-years saved, favouring long-term survival. Lastly, the third-order criterion was prioritization of those with higher social utility, such as healthcare and social service workers.

#### Decision-maker and process

In Thailand, according to the Medical Profession Act 1982 (B.E. 2525), the decisions on a patient’s prognosis and treatment can only be made by the patients’ attending physician. However, in the situation of scarce critical care resources, to alleviate stress and ethical dilemmas faced by physicians, the formation of a triage committee was proposed to assist physicians on allocation decisions. The committee of three healthcare professionals, such as a physician, nurse and/or technical expert, has the primary responsibility to apply the prioritization criteria to each patient upon consideration of ICU admission and reassessment every 48 h and advise physicians on the decision to give, to continue critical care or step down care, such as to palliative care facilities.

### Appeal and documentation mechanisms

The documentation of all patients’ assessment results and decision allocation during the pandemic is necessary for transparency. In addition, a two-step mechanism for appeals to be made by patients, relatives or legal representatives was proposed for the stakeholders to consider: firstly, an immediate appeal of prior allocation decisions for cases where a decision is disputed by the health facility. Secondly, this can be followed by a review by an established review committee to verify disputed decisions through a majority vote.

#### Implementation and enforcement

To ensure the guideline is utilized in appropriate circumstances, the guideline was proposed to be triggered when only 10–20% of critical care resources remain available and to be applied to all patients requiring critical care resources, both patients affected by the pandemic or those with unrelated conditions. The guideline was proposed to be endorsed and issued as a legal document by Medical Council of Thailand, to ensure consistent application across all public and private facilities in Thailand and to provide legal protection to medical doctors who adhere to it.

### Key stakeholder concerns and considerations

During the consultations, five major concerns were identified: (1) ethical principles, (2) criteria to be used for prioritization, (3) decision-makers and the decision-making process, (4) transparency and process of appeal, and (5) implementation and enforcement.

#### Ethics

At both rounds of expert consultations, considerable time was spent discussing the ethical principles that the Thai guideline should comply with. Experts compared the COVID-19 pandemic with wartime when field resources were scarce, and the military goal was for the greatest utility of the society. Applying this utilitarian ideology can be at direct odds with medical ethics, beneficence and non-maleficence principles that health workers practised in normal situations with sufficient resources. Without clear and motivated communication between doctors and patients, the application of “rationing” resources may trigger high levels of stress in some health staff as well as the patients and their relatives. Both stakeholder groups emphasized the need for citizen awareness initiatives to accompany the guideline. Pre-counselling and informing citizens of the need to potentially allocate critical care resources, prior to the implementation of the guide, was recommended to gain a high level of acceptance and limit distrust by the population.

#### Prioritization criteria

The inclusion of age and social values as prioritization criteria was highly contentious in the consultations. Unlike many countries, including Belgium, Italy and the United States [11, 23–25], which use patient age as a prioritizing criterion, most Thai stakeholders did not support an age criterion, even in the form of number of life-years saved. Number of life-years saved, calculated by life expectancy at birth minus patient age, was seen as linked directly to patient age and also gender given females have higher average life expectancy at birth than males [26]. Therefore, the second-order criterion was rejected on the basis that it would contradict the nondiscriminatory basis that health professionals are required to adhere to.

Similarly, the third-order criterion on social usefulness greatly concerned both groups of stakeholders. Many argued that while the criterion itself carries good justification, to compare social values of individuals is not feasible, and decisions can be arbitrary, which may lead to public distrust. Stakeholders from the Buddhist and Muslim communities felt that all individuals hold their own intrinsic value (either for their own family or to the society), and this cannot be compared with one another. It is worth noting that stakeholders preferred all criteria to be objective and quantifiable to enable a transparent and verifiable decision-making process.

#### Decision-makers

A fundamental reason behind establishing a rationing protocol is to alleviate the psychological burden placed on frontline health workers who would be otherwise tasked with deciding who receives potentially lifesaving resources [27, 28]. While stakeholders acknowledged that most guidelines spare attending physicians from making allocation decisions and assign that role to a designated staff or a triage team, stakeholders agreed that in accordance with the Thai medico-legal context, decisions on diagnosis and treatment for patients are to be made by attending physicians. Stakeholders also agreed with the need for a committee to play an advisory role to the attending physicians. However, the “triage team” originally proposed was amended to “Patient Review Committee”, as the word “triage” may carry a negative connotation in Thai society. Medical stakeholders suggested the committee consist of five other healthcare professionals (such as physicians, nurses or relevant experts). The civil society and religious group highlighted that a trusted religious or community leader in the Patient Review Committee would also increase the likelihood of patients and relatives accepting the decisions made, though a counterargument was made that a religious leader in the committee may not be applicable to multi-religious communities. It was agreed that a highly respected member of the community should be selected as a committee member.

#### Appeal mechanisms

Whether or not to establish an appeal mechanism was diligently discussed. On the one hand, allowing patients or representatives to appeal immediately should they disagree with the decision was seen as recognizing patients’ voices and helping ensure objectivity. On the other hand, stakeholders found that an established appeal process could result in delays in decisions, decrease trust in the process and may add an element of unfairness. Stakeholders raised a concern that patients’ ability to appeal is also linked to socioeconomic class. Civil society and social science stakeholders were in favour of having an appeal committee and procedures, while the law stakeholders and medical experts were not, due to the added pressure put on attending physicians. A consensus was finally reached not to establish an appeal committee. Instead, clear and motivated communication between physicians and patients or their relatives about the process and decisions must be actively practised. Regular communication between the physician and the patient’s relatives to update on clinical progress or deterioration as well as prognosis prevents misunderstanding, and boosts trust in the objectivity of clinical decisions and use of critical care resources. In such cases, an appeal process seems unnecessary. Further, there is a high level of trust between doctors and patients, particularly in the public sector [29]. Patient trust also increases when almost all public and private hospitals have gone through an accreditation processes, being accredited or re-accredited by the Healthcare Accreditation Institute [30–32]. In addition, it was determined that regular review of the guideline and a transparent patient registry of what decisions are made was important to allow for future evaluation of the guide to ensure that it fits its purpose.

#### Implementation and enforcement

Although existing guidelines state that guides should be implemented only when demand exceeds supply, stakeholders stressed that it is difficult for frontline health staff to know exactly when surge capacities are fully mobilized and all needed resources in the country have been occupied. Therefore, medical stakeholders suggested the guideline should only be triggered after all efforts have been made to mobilize resources and demand for the resources continue to exceed the supply capacity. Stakeholders added that only when a national public health emergency has been declared and critical resource have been exhausted should the guideline implementation be prompted. This requires higher-level public health officers to routinely monitor and update the resource situation during a public health emergency.

Another matter relating to guideline implementation is enforcement. Similar to the New York Ventilator Allocation Guideline which flagged a concern that there was no legal protection for health staff who adhered to its guideline [24], stakeholders from the medical field emphasized that a legally binding mechanism would be necessary to support and protect healthcare workers in their decisions. A consensus was reached among all stakeholder groups that the endorsement of the guide by the Medical Council should be sought to ensure harmonized implementation nationwide and adherence to existing laws and practices.

### Guideline finalization

**Fig. 2:**
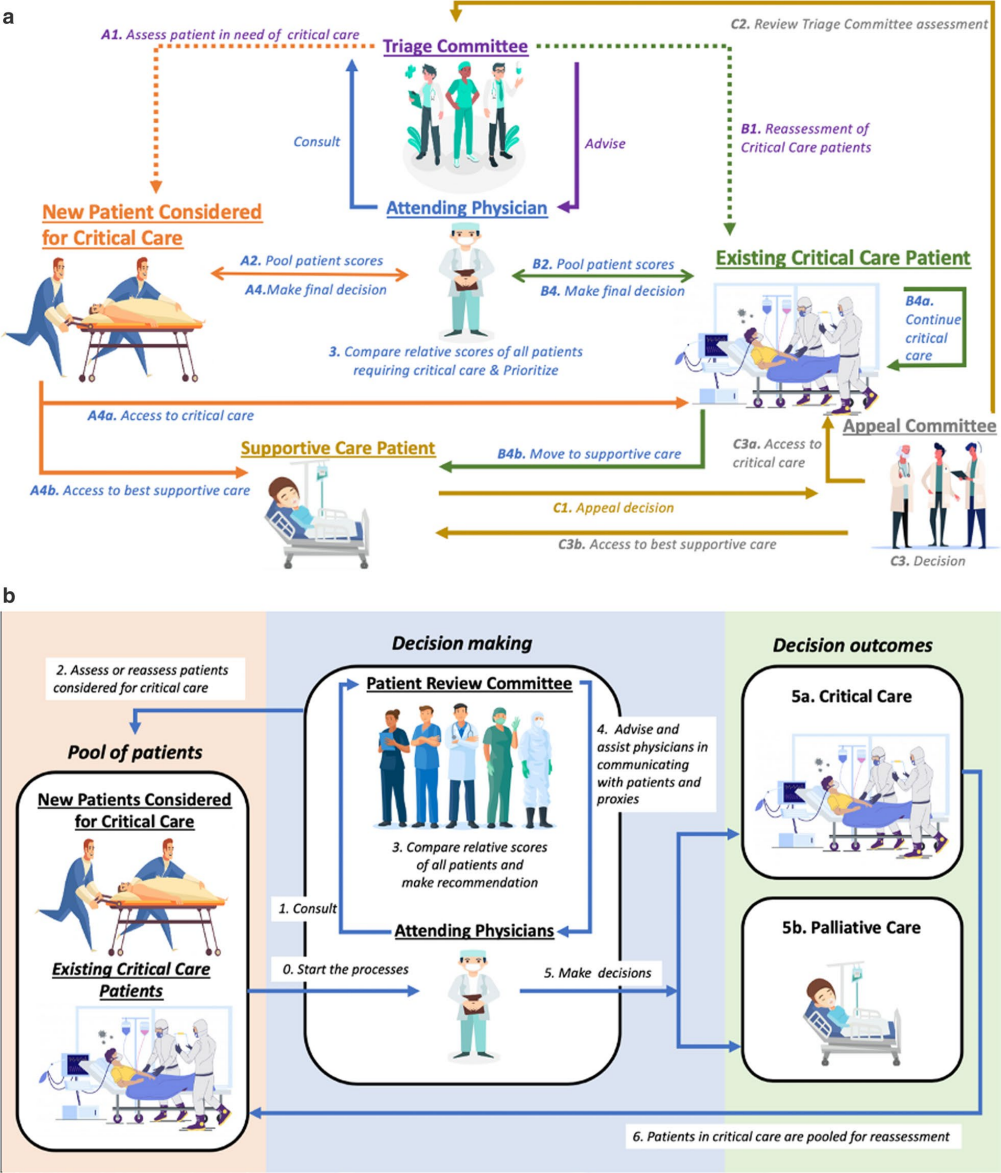
Decision-making steps of critical care resource allocation. **a** Proposed version. **b** Final version after stakeholder consultation

Following two rounds of consultations, a revised guideline was developed by the technical team and circulated to all stakeholders for confirmation and suggested revisions via email if needed. Table 4 summarizes the guideline evolution at different steps of development, and Fig. 2 compares the proposed and final version of decision-making steps.

**Table 4:** Summary of guideline evolution at each development step

Key content	Step 1: rapid review (first draft)	Step 2: key informant interview (second draft)	Step 3: multi-stakeholder consultation (final draft)
Guideline principle	Save the most lives Save the most life-years Benefit to others	Save the most lives Save the most life-years Benefit to others	Utilitarianism: saving the most lives
Prioritization criteria	Apply three-order criteria: Clinical prognosis, e.g. SOFA, CFS; cognitive impairment assessment Number of life-years saved Social usefulness Allocation decisions are based on relative scores No cut-off score is applied	Apply three-order criteria: Clinical prognosis using one or more of the following tools: Charlson Comorbidity Index, SOFA, frailty assessment such as CFS, cognitive impairment assessment Number of life-years saved Social usefulness Allocation decisions are based on relative scores No cut-off score is applied	Assess patients based on clinical prognosis using at least two of the following tools: Charlson Comorbidity Index, SOFA, frailty assessment such as CFS, cognitive impairment assessment Allocation decisions are based on relative scores No cut-off score is applied Each health facility must apply the same sequence of tools consistently across all cases
Application	Applicable to all patients requiring critical care resources Prior to ICU admission Reassessment every 48 hours during ICU stay	Applicable to all patients requiring critical care resources Prior to ICU admission Reassessment every 48 hours during ICU stay	Applicable to all patients requiring critical care resources Prior to ICU admission Reassessment as appropriate during ICU stay
Decision-making	Triage committee of three healthcare professionals advises an attending physician on allocation	Attending physician is a decision-maker Triage committee of three healthcare professionals advises an attending physician on allocation	Attending physician is a decision-maker Patient review committee of five health and non-health experts advises an attending physician on allocation decision and communication with patient and families
Review process	Document assessment result and allocation decisions in a registry Registry information can be reviewed by a staff/team in the hospital who are not involved in the first decision or external expert(s) Appeal mechanism was proposed to be considered	Document assessment results and allocation decisions in a registry Registry information can be reviewed by a staff/team in the hospital who are not involved in the first decision or external expert(s) Appeal mechanism was proposed to be considered	Document assessment results and allocation decisions in a registry Registry information can be reviewed by a staff/team in the hospital who are not involved in the first decision or external expert(s)
Implementation	When only 10–20% of critical care resources remain available	When only 10–20% of critical care resources remain available	National public health emergency AND All efforts have been made to mobilize resources and demand still exceeds supply
Enforcement	The guideline is to be endorsed by the Medical Council of Thailand	The guideline is to be endorsed by the Medical Council of Thailand	The guideline is to be endorsed by the Medical Council of Thailand. Current status of endorsement is unclear due to the pandemic’s changing situation

*SOFA* Sequential Organ Failure Assessment, *CFS* Clinical Frailty Scale

Upon finalization of the guideline content, the guide was presented at a high-level governmental meeting of decision-makers and academics, including executive board members of the Medical Council of Thailand. The importance and the need for the guide was acknowledged. However, the favourable outcomes of COVID-19 containment in the country leading to an average of less than three new daily cases of COVID-19 between 6 and 25 June 2020 resulted in the announcement of the third and final phases, marking the end of lock-down measures in Thailand on 1 July 2020. This has made the endorsement of the guideline no longer a matter of urgency, and therefore it was decided that commencing a legal process with the Medical Council should be put on hold. However, recognizing looming threats of new waves of infection after the unlocking phase, the guide can be adopted should the necessary situation arise.

## Discussion

Clinical practice guidelines must be evidence-informed, to ensure consistency with the best practices of the scientific community, and the development process must involve a wide range of relevant expertise and stakeholders who may be affected by the recommendations to ensure applicability, feasibility and relevance to the situations and contexts [17, 33, 34]. As a result, the process of developing health guidelines often takes time, ranging from 6 months to 3 years to produce [17, 18]. This poses significant challenges for the development of guidelines during public health emergencies, where fast evidence-informed and context-specific recommendations are required.

In the context of the COVID-19 pandemic, especially at the peak of the outbreak where resources were being quickly depleted, there was uncertainty in depleting resources for which the allocation recommendations may have been needed. Additionally, in Thailand, as with many countries, the situation in which some lives have to be sacrificed due to insufficient resources is alien for many health professionals; therefore, guidance on how to make these stark decisions is essential. The idea of “rationing” healthcare is an immensely sensitive issue and may cause alarm and foster unconstructive discourse in the general public. Therefore, the guideline development process must be fast while maintaining methodological and scientific rigor, transparent, suitable to medico-legal context and be carefully planned to aid public acceptance. The development of Thai guideline aimed for evidence-based and context-relevant recommendations to be executed within a short time frame [18].

First, we applied a rapid review within a month’s time frame to ensure recommendations were based on the best available evidence [17, 18]. Although guidelines ideally apply a full systematic review to inform recommendations, rapid reviews can provide adequate advice for both clinical and policy decisions and inform actions in emergency situations when a full systematic review is not feasible [17, 18]. Defined as an accelerated or modified systematic review method, rapid reviews apply a more targeted scope and search criteria, while allowing for modifications in search strategy as the evidence base is explored, though remaining systematic, transparent and explicit in both the identification and use of evidence [17, 18].

Similar to the 2014 Ebola outbreak in West Africa, where no relevant data could be obtained through systematic review of published literature [17], we found limited relevant information using standard systematic search methods. Therefore, adapting our rapid review strategy and searching in the International Society for Priorities in Health website for both academic and grey literature and existing national and international guidelines, and explicitly specifying inclusion and exclusion criteria, was most applicable.

Second, we applied a qualitative research methodology, using key informant interviews and stakeholder consultations, to ensure adaptation of the recommendations for acceptability, feasibility and stakeholder buy-in [17]. Due to the absence of guidelines from low- and middle-income countries (LMICs), most LMICs resort to international guidelines or those developed by high-income countries, which may be less likely to be accepted or successfully implemented because of the differences in health systems and cultural and social norms [35, 36].

The key informant interviews allowed us to compare best practices from the rapid review with current norms and practices of physicians in caring for COVID-19 and other ICU patients in Thailand. This is important, because less variance from the current practice means less investment in capacity-building interventions for healthcare workers, especially in the urgent COVID-19 situation. Also, we confirmed the lack of existing protocols and guidelines in Thailand: to prevent duplication, the key informants confirmed the need for a COVID-19-specific guide.

Stakeholders were invited from a mix of constituencies, while also pursuing a gender balance. Of the 21 stakeholders participating in the two rounds of hearings, seven were female (33%). Because all four religious leaders were male, it was difficult to achieve a gender balance. Physicians contribute to the scientific evidence and real-life practice on rationing; these are palliative and respiratory care and emergency medical experts who make daily decisions on the use of critical care resources. The three CSO representatives have a long-standing track record on safeguarding public interests, such as the Foundation for Consumers, CSO representatives in the National Health Security Board and a support group for palliative patients.

The engagement of stakeholders has become an essential component of guideline development and implementation to improve acceptability and address potential concerns such as inequities in recommendations among varying populations [37]. Stakeholder consultations have been utilized in the development of similar guidelines for scarce resource allocation in other countries, including the United States and Canada [38, 39]. There are four levels of stakeholder engagement: communication, consultation, collaboration and coproduction [37]. In our process, we aimed to engage with stakeholders on two levels: consultation to gather stakeholder views, feedback and experiences; and collaboration, through which stakeholders can influence the decisions on the guideline content. We used a consensus approach to decision-making on key issues of each recommendation in the guide, a process strongly encouraged for WHO’s guideline development groups [17]. Multi-stakeholder engagement allowed recommendations to be adapted to be medically sound, applicable to existing laws and practices and acceptable to citizens, including majority and minority populations.

Given the time and resource constraints during the COVID-19 state of emergency and travel restrictions in Thailand at the time of data collection, the technical team was only able to interview two ICU physicians from public hospitals in Bangkok city. As a result, a limitation we faced was not being able to physically visit facilities, obtain information on practices more representative of the whole country and of practices in private facilities. Not only expediency issues, but urgency to conclude the national guideline prevented a full-blown and thorough public consultation with all stakeholders, though representatives from CSOs had reflected and safeguarded the interests of citizens and patients. In the second round of consultation with policy-makers and social sciences stakeholders, we invited three out of the 11 CSO stakeholder groups. From our observations, the religious leaders and scholars, as well as the CSOs, had reflected the concerns and interests of the society, patients and citizens very well.

Further, in engaging with stakeholders, we were unable to organize consultations with a large number of participants due to social distancing measures. As some stakeholders did not find teleconferencing to be convenient, we selected a mixed consultation approach, allowing options for both in-person or teleconference participation. This allowed us to engage with a more diverse group of stakeholders from throughout the country, who would otherwise not have been able to participate. To ensure all voices were equally heard, a capable moderator was necessary, to call on various stakeholder groups, regardless of whether they were attending in person or remotely.

## Conclusion

The attempt to develop critical care resource allocation guideline provides useful lessons for policy-makers, public health practitioners, professional bodies and academic scholars in Thailand and other LMICs that may find it necessary to establish a similar allocation guideline. Reviews of international experience are important to provide solid scientific ground for the content of the guideline to be adapted in line with the country context. Multi-sectoral stakeholder consultation provides constructive comments and contextualized views to adapt international experience to local contexts. To the best of our knowledge, the process of developing a rapid national guideline for rationing of resources during the COVID-19 pandemic, particularly in LMIC settings, has not yet been shared or publicly available. Therefore, this paper is among the first of its kind, and can provide an insight for the development of similar guidelines, especially in other LMICs. We recommend the guideline goes through a full-blown public consultation once the COVID-19 situation eases to allow more active participation and interaction.

## Data Availability

Not applicable.

## Abbreviations

COVID-19: Coronavirus disease 2019
CSF: Clinical Frailty Scale
FAST: Functional Assessment Staging Test
HITAP: Health Intervention and Technology Assessment Program
ICU: Intensive care unit
IHPP: International Health Policy Program
LMICs: Low- and middle-income countries
Modified IQ CODE: Modified Informant Questionnaire on Cognitive Decline in the Elderly
MOPH: Ministry of Public Health
NHCO: National Health Commission Office
SOFA: Sequential Organ Failure Assessment

## References

[1] Cucinotta D, Vanelli M (2020). WHO Declares COVID-19 a Pandemic. Acta Bio Medica Atenei Parmensis.

[2] World Health Organization. Coronavirus (COVID-19) Dashboard [Internet]. 2020. https://covid19.who.int/. Accessed 21 Jul 2020.

[3] Emanuel EJ, Persad G, Upshur R, Thome B, Parker M, Glickman A, et al. Fair Allocation of Scarce Medical Resources in the Time of Covid-19. N Engl J Med [Internet]. 2020. http://www.nejm.org/doi/10.1056/NEJMsb2005114. Accessed 1 May 2020. 10.1056/NEJMsb2005114 32202722

[4] Rosenbaum L. Facing Covid-19 in Italy — Ethics, Logistics, and Therapeutics on the Epidemic’s Front Line. N Engl J Med [Internet]. 2020. http://www.nejm.org/doi/10.1056/NEJMp2005492. Accessed 1 May 2020. 10.1056/NEJMp2005492 32187459

[5] Maves RC, Downar J, Dichter JR, Hick JL, Devereaux A, Geiling JA (2020). Triage of Scarce Critical Care Resources in COVID-19 An Implementation Guide for Regional Allocation. Chest.

[6] Arya A, Buchman S, Gagnon B, Downar J (2020). Pandemic palliative care: beyond ventilators and saving lives. CMAJ.

[7] White DB, Lo B (2020). A framework for rationing ventilators and critical care beds during the COVID-19 pandemic. JAMA.

[8] World Health Organization. Ethics and COVID-19: resource allocation and priority-setting [Internet]. https://www.who.int/ethics/publications/ethics-and-covid-19-resource-allocation-and-priority-setting/en/. Accessed 1 Apr 2020.

[9] Swiss Academy of Medical Sciences (2020). COVID-19 pandemic: triage for intensive-care treatment under resource scarcity. Swiss Med Wkly..

[10] National Institute for Health and Care Excellence, United Kingdom National Health Service. COVID-19 rapid guideline: critical care in adults [Internet]. 2020. https://www.nice.org.uk/guidance/ng159. Accessed 1 Apr 2020.

[11] Belgian Society of Intensive Care Medicine. Ethical principles concerning proportionality of critical care during the 2020 COVID-19 pandemic in Belgium: advice by the Belgian Society of Intensive care medicine [Internet]. http://www.siz.be/wp-content/uploads/COVID-19-ethical_final_c.pdf. Accessed 1 Apr 2020.

[12] Deutschen Interdisziplinären Vereinigung für Intensiv- und Notfallmedizin. Entscheidungen über die Zuteilung von Ressourcen in der Notfallund der Intensivmedizin im Kontext der COVID-19-Pandemie [Internet]. 2020. https://www.divi.de/empfehlungen/publikationen/covid-19/1540-covid-19-ethik-empfehlung-v2/file. Accessed 1 Apr 2020.

[13] World Health Organization. WHO statement on novel coronavirus in Thailand. 2020; https://www.who.int/news-room/detail/13-01-2020-who-statement-on-novel-coronavirus-in-thailand.. Accessed 1 Apr 2020.

[14] Okada P, Buathong R, Phuygun S, Thanadachakul T, Parnmen S, Wongboot W (2020). Early transmission patterns of coronavirus disease, 2019 (COVID-19) in travellers from Wuhan to Thailand, January 2020. Eurosurveillance.

[15] Department of Disease Control, Ministry of Public Health. Covid-19 Infected Situation Reports [Internet]. 2020. https://covid19.ddc.moph.go.th/en. Accessed 21 Jul 2020.

[16] Department of Medical Sciences. List of hospitals and resources for patients Covid-19 [Internet]. http://cov19bkkrm.dms.go.th/covid/. Accessed 1 Apr 2020.

[17] World Health Organization (2014). WHO handbook for guideline development.

[18] Kowalski SC, Morgan RL, Falavigna M, Florez ID, Etxeandia-Ikobaltzeta I, Wiercioch W (2018). Development of rapid guidelines: 1. Systematic survey of current practices and methods. Health Res Policy Sys.

[19] Rajan D, Mathurapote N, Putthasri W, Posayanonda T, Pinprateep P, de Courcelles S (2019). Institutionalising participatory health governance: lessons from nine years of the national health assembly model in Thailand. BMJ Glob Health.

[20] Kramer JB, Brown DE, Kopar PK (2020). Ethics in the time of coronavirus: recommendations in the COVID-19 pandemic. J Am Coll Surg..

[21] Ramanathan K, Antognini D, Combes A, Paden M, Zakhary B, Ogino M (2020). Planning and provision of ECMO services for severe ARDS during the COVID-19 pandemic and other outbreaks of emerging infectious diseases. Lancet Respir Med..

[22] Tricco AC, Langlois EV, Straus SE. Rapid reviews to strengthen health policy and systems: a practical guide. 2017. 10.1136/bmjgh-2018-001178 PMC6407563 30899562

[23] Riccioni L, Bertolini G, Giannini A, Vergano M, Gristina G, Livigni S (2020). Raccomandazioni di etica clinica per l’ammissione a trattamenti intensivi e per la loro sospensione, in condizioni eccezionali di squilibrio tra necessità e risorse disponibili. Recenti Prog Med.

[24] New York State Department of Health, New York State Task Force on Life and the Law. Ventilator Allocation Guidelines [Internet]. 2015. https://www.health.ny.gov/regulations/task_force/reports_publications/docs/ventilator_guidelines.pdf. Accessed 11 Jun 2020

[25] University of Pittsburgh. Allocation of Scarce Critical Care Resources During a Public Health Emergency [Internet]. 2020. https://ccm.pitt.edu/sites/default/files/UnivPittsburgh_ModelHospitalResourcePolicy_2020_04_15.pdf. Accessed 1 Apr 2020.

[26] World Bank. World Bank Development Indicators [Internet]. http://datatopics.worldbank.org/world-development-indicators/. Accessed 1 Apr 2020.

[27] Lai J, Ma S, Wang Y, Cai Z, Hu J, Wei N (2020). Factors associated with mental health outcomes among health care workers exposed to coronavirus disease 2019. JAMA Netw Open.

[28] Pappa S, Ntella V, Giannakas T, Giannakoulis VG, Papoutsi E, Katsaounou P (2020). Prevalence of depression, anxiety, and insomnia among healthcare workers during the COVID-19 pandemic: a systematic review and meta-analysis. Brain Behav Immun..

[29] Riewpaiboon W, Chuengsatiansup K, Gilson L, Tangcharoensathien V (2005). Private obstetric practice in a public hospital: mythical trust in obstetric care. Soc Sci Med.

[30] James PTJ (2012). The impact of medical tourism on Thai private hospital management: informing hospital policy. Glob J Health Sci.

[31] Smits H, Supachutikul A, Mate KS (2014). Hospital accreditation: lessons from low- and middle-income countries. Global Health.

[32] Mansour W, Boyd A, Walshe K (2020). The development of hospital accreditation in low- and middle-income countries: a literature review. Health Policy Plan.

[33] Oxman AD, Fretheim A, Schünemann HJ (2006). Improving the use of research evidence in guideline development: introduction. Health Res Policy Sys..

[34] Schünemann HJ, Fretheim A, Oxman AD (2006). Improving the use of research evidence in guideline development: 13. Applicability, transferability and adaptation. Health Res Policy Sys..

[35] Mehndiratta A, Sharma S, Gupta NP, Sankar MJ, Cluzeau F (2017). Adapting clinical guidelines in India—a pragmatic approach. BMJ.

[36] Olayemi E, Asare EV, Benneh-Akwasi Kuma AA (2017). Guidelines in lower-middle income countries. Br J Haematol.

[37] Petkovic J, Riddle A, Akl EA, Khabsa J, Lytvyn L, Atwere P (2020). Protocol for the development of guidance for stakeholder engagement in health and healthcare guideline development and implementation. Syst Rev [Internet].

[38] Daugherty Biddison EL, Faden R, Gwon HS, Mareiniss DP, Regenberg AC, Schoch-Spana M (2019). Too Many Patients…A Framework to Guide Statewide Allocation of Scarce Mechanical Ventilation During Disasters. Chest [Internet].

[39] Christian MD, Hawryluck L, Wax RS, Cook T, Lazar NM, Herridge MS (2006). Development of a triage protocol for critical care during an influenza pandemic. Can Med Assoc J.

[40] Österreichischen Gesellschaft für Anästhesiologie, Reanimation und Intensivmedizin. Allokation intensivmedizinischer Ressourcen aus Anlass der Covid-19-Pandemie [Internet]. https://www.oegari.at/web_files/cms_daten/covid-19_ressourcenallokation_gari-statement_v1.7_final_2020-03-17.pdf. Accessed 1 Apr 2020.

[41] British Medical Association. COVID-19—ethical issues. A guidance note [Internet]. https://www.bma.org.uk/media/2360/bma-covid-19-ethics-guidance-april-2020.pdf. Accessed 1 Apr 2020.

[42] The Hastings Center. Ethical Framework for Health Care Institutions & Guidelines for Institutional Ethics Services Responding to the Coronavirus Pandemic [Internet]. 2020. https://www.thehastingscenter.org/ethicalframeworkcovid19/. Accessed 1 Apr 2020.

[43] World Health Organization (2016). Guidance for managing ethical issues in infectious disease outbreaks.

